# A general model for determination of molecular structures of anti-biofilm compounds through metric-based resolvability

**DOI:** 10.1038/s41598-026-51717-2

**Published:** 2026-05-06

**Authors:** Asma Alasmri, Nor Muhainiah Mohd Ali, Ali Ahmad, Muhammad Faisal Nadeem

**Affiliations:** 1 Department of Mathematical Sciences, Faculty of Science, Universiti Teknologi Malaysia, Johor Bahru, 81310 UTM Johor Malaysia; 2 Department of Mathematics, College of Science, Jazan University, Jazan, Saudi Arabia; 3 Department of Computer Science, College of Engineering and Computer Science, Jazan University, Jazan, Saudi Arabia; 4 Department of Mathematics, COMSATS University Islamabad, Lahore Campus, Lahore, Pakistan

**Keywords:** Anti-biofilm drugs, Molecular graph, Metric dimension, Structural descriptors, Chemistry, Computational biology and bioinformatics, Drug discovery, Mathematics and computing

## Abstract

Based on distance measures descriptors can indeed provide useful information about structural aspects of a molecule by providing a unique identification of atoms for a particular molecular graph. A set of vertices that allows the atoms of a graph to be uniquely identified based on their distances to the set is called a resolving set, whereas the smallest such set is called the metric dimension of the graph. In this research work, we discuss the metric dimension of the molecular graph of some of the most influential anti-biofilm agents such as chlorhexidine, colistin, berberine, usnic acid, ellagic acid, curcumin, and epigallocatechin gallate. The obtained results show that metric dimension distinguishes these compounds according to their topological complexity, degree of branching, and structural repetition, and therefore provides a useful distance-based descriptor for molecular characterization and graph-based identification of anti-biofilm agents.

## Introduction

Biofilm-based infections are an important challenge for biomedical chemistry because of their remarkable tolerance against antimicrobials and standard therapeutic regimens. Biofilm formation was historically documented during the seventeenth century when Antonie van Leeuwenhoek, using his homemade microscope, identified dental plaque as an ordered microbial aggregate^1^. Follow-up studies have revealed that biofilms are very well-organized microbial populations encapsulated in an extracellular matrix that is secreted from within^2^. It is based on this understanding that there has been an investigation of a wide range of compounds with biofilm-inhibiting properties.

The classes of molecules which have been researched for anti-biofilm properties to the highest degree are synthetic molecules, especially chlorhexidine and colistin, and natural molecules like berberine, usnic acid, ellagic acid, curcumin, and epigallocatechin gallate. Such molecules belong to different classes of chemicals and have different molecular structures. Their anti-biofilm properties have been linked with several actions: inhibition of biofilm formation and disruption. For instance, usnic acid is a lichen-derived secondary compound with established anti-biofilm properties, which was shown to inhibit biofilm formation as well as disrupt pre-existing biofilms, where its rigid polycyclic skeleton and high density of hydroxyl and oxygen atoms play a critical role in inhibiting biofilm formation^3^.

The biological response to anti-biofilm agents is strongly influenced by the basic molecular structure of the agent itself. Pamp et al. (2008) have shown that biofilm tolerance to colistin in Pseudomonas aeruginosa biofilms is related to metabolically active populations, and its regulation is dependent on the pmr operon and the mexAB-oprM efflux system, emphasizing that biofilm tolerance is generally related to molecular interactions at a chemical level^4^. Blanco et al. found that sub-inhibitory concentrations of epigallocatechin gallate caused significant reductions in slime production and biofilm formation by ocular *Staphylococcus* isolates, and noted the importance of polyphenolic structure to anti-biofilm properties^5^.

In addition to the classical antimicrobial properties, recent research has emphasized the need for non-microbicidal approaches to control biofilms. Stowe et al. (2011) have discussed anti-biofilm approaches based on the use of small molecules of marine sponge origin that can either inhibit biofilm or cause dispersal of existing biofilms without killing microbes^6^. In a related stream of research, Qvortrup et al. have proposed a mechanism-based approach to the rational design of small-molecule anti-biofilm compounds, focusing on inhibitors of early adhesion mechanisms, quorum sensing, and c-di-GMP pathways. Taken together, these investigations highlight the crucial importance of molecular structure for anti-biofilm activity^7^.

From the point of view of chemical modeling, the application of graph theory provides an objective mathematical tool for the description of the molecular structure and the extraction of quantitative parameters. In the field of chemistry, molecules are represented as graphs, where the vertices are associated with the atoms, and the edges with the chemical bonds that link them. Based on the concept of graph theory, the graph theoretical descriptors are widely accepted and named topological indices, enabling the application of QSAR and QSPR for the prediction of the biological activity and physicochemical properties of compounds solely on the basis of the molecular structure. Recently, Ahmed et al.^8^ presented the application of the QSPR approach via graph theory for the discovery of anti-biofilm drugs, combining the topological indices, generated by computational methods and regression techniques for the establishment of quantitative relationships between the molecular structure and anti-biofilm activity.

Within the distance-based graph descriptors, the metric dimension has a special position owing to its capacity to describe vertex uniqueness in a graph. A resolving subset of vertices in a graph is a subset of vertices that uniquely identifies all other vertices in the graph using their distances from that subset of vertices, and the minimum-size subset of vertices that is a resolving subset of vertices defines the metric dimension of the graph. Regarding molecular graph structures, the metric dimension of the graph describes the efficiency with which the vertices in the molecular graph are differentiated on the basis of their intervertex distances.

Inspired by the chemical diversity and biological significance of anti-biofilm compounds, we examine the metric dimension of molecular graphs for chlorhexidine, colistin, berberine, usnic acid, ellagic acid, curcumin, and epigallocatechin gallate. Through a systematic analysis and comparison of the metric dimension values of these compounds, the goal is to validate and affirm the utility of the metric dimension as a structurally significant tool in chemical graph theory.

### Metric dimension

Metric dimension is a distance invariant in graphs and represents a numerical value corresponding to the minimum number of vertices required to code all other vertices uniquely with their distance representations. The idea was proposed independently in the 1970s by Slater and later by Harary and Melter. The idea was first proposed by Slater in facility location problems and is noted as locating sets or resolving sets in Ref. 9, while its basic properties as a metric dimension are proposed by Harary and Melter in Ref. 10. Using their terminology, for any connected graph *G*, the metric dimension $\dim (G)$ is then said to be the size of a minimum resolving set. Since then, metric dimension has found considerable use as a graph parameter, especially in chemical graph theory, where distance matrices help to encode the location of atoms in graphs that model molecules.

From a computational complexity perspective, Garey et al.^11^ proved that finding the metric dimension of a graph is an NP-complete problem. The research has important implications and has been applied to various fields of science and engineering, such as pharmaceutical chemistry^12^, image recognition^13^, and classical false coin problems^14^, apart from computer and communication network problems^15^, and has been studied in the framework of the branch of graph theory titled combinatorial optimization^16^ for graphical representations of navigation and landmark-based localization^17^ problems. SebHo and Tannier^16^ studied the graph’s metric generators and their solutions for the problems of graph search, false coin problems, and the concepts of connected joins, apart from tackling the computational issues and graph optimization.

Sharma et al.^18^, for example, focused on the metric and edge metric dimensions, as well as the topological parameters of *H*1*N*1 antiviral drug molecules, and found that these dimensions have become an essential framework in predicting molecular properties and, hence, in improving pharmaceutical models using the QSPR approach, whereas Liu et al.^19^ discussed the metric and fault-tolerant metric dimensions of the GeSbTe superlattices and found that these metrics are useful in identifying networks that optimize molecular and electronic systems. The application of the metric value of drug molecular graphs and their efficiency for differentiating the atomic coordinates was discussed by Singh et al.^20^, and the vertex and edge resolvability of different drug graph topologies was further studied by Kiran et al.^21^, proving the effectiveness of the metric value for differentiating the graphs. Continuing to the complex topological structure of graphs, the application of the metric value for Möbius-type geometric graphs and benzenoid systems was studied by Hayat et al.^22^, addressing the issues of computational complexity. Mhagama et al.^23^ demonstrated the efficacy of edge resolvability of the line graphs of anti–viral drugs for modeling the structure required for the design of an Mpox drug molecule, whereas Farooq et al.^24^ studied the metric and edge metric dimension of $SiO_{2}$ nanosheets, nanotubes, and nanotori for design As for investigation, Gu et al.^25^ demonstrated that the metric dimensions of hypoglycemic drug molecules are successful at identifying subtle structural differences for effective graph-based molecular modeling, whereas Pandeeswari and Sankar^26^ proved that metric-dimensional as well as edge metric-dimensional resolvability of breast cancer drug graphs facilitates structural differentiation for effective design/formulation of drugs.

#### Definition 1.1

Let *G* be a finite, simple, and connected graph with vertex set *V*(*G*) and edge set *E*(*G*). For any two vertices $u,v \in V(G)$, the distance between *u* and *v* is denoted by $d_G(u,v)$, and is defined as the length of a shortest *u*–*v* path in *G*. Whenever the underlying graph is clear from the context, we simply write *d*(*u*, *v*) instead of $d_G(u,v)$.

#### Definition 1.2

Let *G* be a connected graph, and let $S=\{s_1,s_2,\dots ,s_k\}\subseteq V(G)$ be an ordered subset of vertices. Then the metric representation of a vertex $x\in V(G)$ with respect to *S* is defined by

$$r_G(x\mid S)=\big (d_G(x,s_1),d_G(x,s_2),\dots ,d_G(x,s_k)\big ).$$

When the graph *G* is clear from the context, we write $r(x\mid S)$ instead of $r_G(x\mid S)$.

#### Definition 1.3

A subset $S\subseteq V(G)$ is called a resolving set of *G* if for every pair of distinct vertices $x,y\in V(G)$, we have

$$r_G(x\mid S)\ne r_G(y\mid S).$$

The minimum cardinality of a resolving set of *G* is called the metric dimension of *G*, and is denoted by

$$\dim (G).$$

#### Definition 1.4

Let *G* be a connected graph and let $v \in V(G)$. For a positive integer *k*, the distance-*k* neighborhood of *v* is defined by

$$N_k(v)=\{u \in V(G): d(u,v)=k\}.$$

For each molecular graph considered in this work, we use the symbols $ChD_{34}$, $CoD_{81}$, $BeD_{25}$, $UaD_{25}$, $EaD_{22}$, $CuD_{27}$, and $EgD_{33}$ consistently to denote the corresponding hydrogen-suppressed molecular graphs. Accordingly, their vertex and edge sets are written as $V(ChD_{34})$, $E(ChD_{34})$, and similarly for the remaining graphs.

## Metric dimension of the chlorhexidine drug

**Fig. 1:**
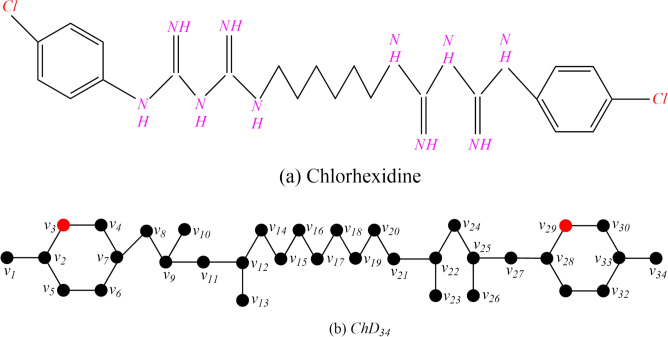
(**a**) Chemical structure of chlorhexidine. (**b**) Labeled hydrogen-suppressed molecular graph $ChD_{34}$ of chlorhexidine.

Chlorhexidine^27^, denoted by $ChD_{34}$, is a well-known broad-spectrum antimicrobial and anti-biofilm compound with the molecular formula $C_{22}H_{30}Cl_{2}N_{10}$. In pharmaceutical and clinical applications, it is predominantly used in the form of Chlorhexidine gluconate, which is obtained by combining the parent molecule with two gluconic acid units, represented as $2C_{6}H_{12}O_{7}$. Owing to its structural complexity and therapeutic relevance, Chlorhexidine serves as an important model molecule for graph-theoretic analysis. The IUPAC name of Chlorhexidine is $N,N''''$-hexane-1,6-diylbis[$N'$-(4-chlorophenyl)(imidodicarbonimidic diamide)]. The molecular graph representation and the corresponding chemical structure of $ChD_{34}$ are illustrated in Fig. 1a and b, respectively.

### Theorem 2.1

*Let* $ChD_{34}$ *denote the molecular graph of the drug chlorhexidine. Then* $\dim (ChD_{34})=2$.

### Proof

We regard $ChD_{34}$ as the hydrogen-suppressed heavy-atom graph of chlorhexidine; vertices represent non-hydrogen atoms and edges represent covalent bonds. Since $ChD_{34}$ contains cycles and is not a path, $\dim (ChD_{34})\ge 2$ (indeed, the only connected graphs of metric dimension 1 are paths).

We now exhibit a resolving set of size 2. Choose two landmarks on opposite terminal aryl rings, indexed in our labelling as $v_3$ (on the left ring) and $v_{29}$ (on the right ring). Consider the ordered pair of distances

$$r(v_\xi \mid S)=\bigl (d(v_\xi ,v_{3}),\,d(v_\xi ,v_{29})\bigr ) \quad \text {with}\quad S=\{v_{3},v_{29}\},\ \ 1\le \xi \le n=|V(ChD_{34})|.$$

Intuitively, any path from the left to the right half of the molecule must traverse the same sequence of articulations (ring $\rightarrow$ linker $\rightarrow$ central chain $\rightarrow$ linker $\rightarrow$ ring). Consequently, as one walks the heavy-atom backbone from left to right, $d(\,\cdot ,v_3)$ is (piecewise) nondecreasing while $d(\,\cdot ,v_{29})$ is (piecewise) nonincreasing, with discrete ”jumps” at block boundaries. This opposition of trends is what will guarantee unique distance pairs.

A direct computation along the molecular graph carried out by accumulating shortest-path lengths from each vertex to the two landmarks as one moves block-by-block through the structure yields the following case-wise formulas:

$$\text {for }\xi =1,\ldots ,4:\quad r(v_\xi \mid S)=\Bigl (3-\xi +2\Bigl \lfloor \frac{\xi -1}{3}\Bigr \rfloor ,\ n-\xi -11\Bigr );$$

$$\text {for }\xi =5,\ldots ,9:\quad r(v_\xi \mid S)=\Bigl (\xi -3-2\Bigl \lfloor \frac{\xi -4}{3}\Bigr \rfloor ,\ n-\xi -9\Bigr );$$

$$\text {for }\xi =10,\ldots ,15:\quad r(v_\xi \mid S)=\Bigl (\Bigl \lfloor \frac{\xi +5}{3}\Bigr \rfloor +\Bigl \lfloor \frac{\xi -9}{3}\Bigr \rfloor ,\ n-\xi -7-\Bigl \lfloor \frac{\xi -8}{3}\Bigr \rfloor +2\Bigl \lfloor \frac{\xi -10}{3}\Bigr \rfloor \Bigr );$$

$$\text {for }\xi =16,\ldots ,23:\quad r(v_\xi \mid S)=\Bigl (\xi -7,\ n-\xi -7+2\Bigl \lfloor \frac{\xi -15}{8}\Bigr \rfloor \Bigr );$$

$$\text {for }\xi =24,\ldots ,26:\quad r(v_\xi \mid S)=\Bigl (\xi -8,\ n-\xi -6+2\Bigl \lfloor \frac{\xi -24}{2}\Bigr \rfloor \Bigr );$$

$$\text {for }\xi =27,\ldots ,30:\quad r(v_\xi \mid S)=\Bigl (\xi -9,\ n-\xi -5+2\Bigl \lfloor \frac{\xi -27}{3}\Bigr \rfloor \Bigr );$$

$$\text {for }\xi =31,\ldots ,n:\quad r(v_\xi \mid S)=\Bigl (\xi -11,\ \xi {-}n+5-2\Bigl \lfloor \frac{\xi -31}{2}\Bigr \rfloor \Bigr ).$$

Each index interval corresponds to a contiguous structural fragment (left ring sector; left linker/biguanide; central aliphatic chain; right linker/biguanide; right ring sector). Within any single interval, both coordinates of $r(v_\xi \mid S)$ are affine in $\xi$ except at finitely many breakpoints governed by the floor terms, and between successive indices $\xi$ and $\xi +1$ at least one coordinate changes by $\pm 1$. Hence, within each interval the map $\xi \mapsto r(v_\xi \mid S)$ is injective.

At the boundaries between intervals, the pair $(d(v_\xi ,v_3),d(v_\xi ,v_{29}))$ acquires an offset because crossing an articulation (ring $\leftrightarrow$ linker or linker $\leftrightarrow$ chain) forces any shortest path to pass through a new gateway vertex. This adds at least 1 to one of the two coordinates in the pair relative to the last value in the preceding interval. Therefore no pair produced at the start of a new interval can coincide with a pair from the interior of the previous interval. A straightforward check of the displayed boundary values confirms that the terminal pair in each interval differs from the initial pair in the next interval in at least one coordinate. $\square$

## Metric dimension of colistin drug

**Fig. 2:**
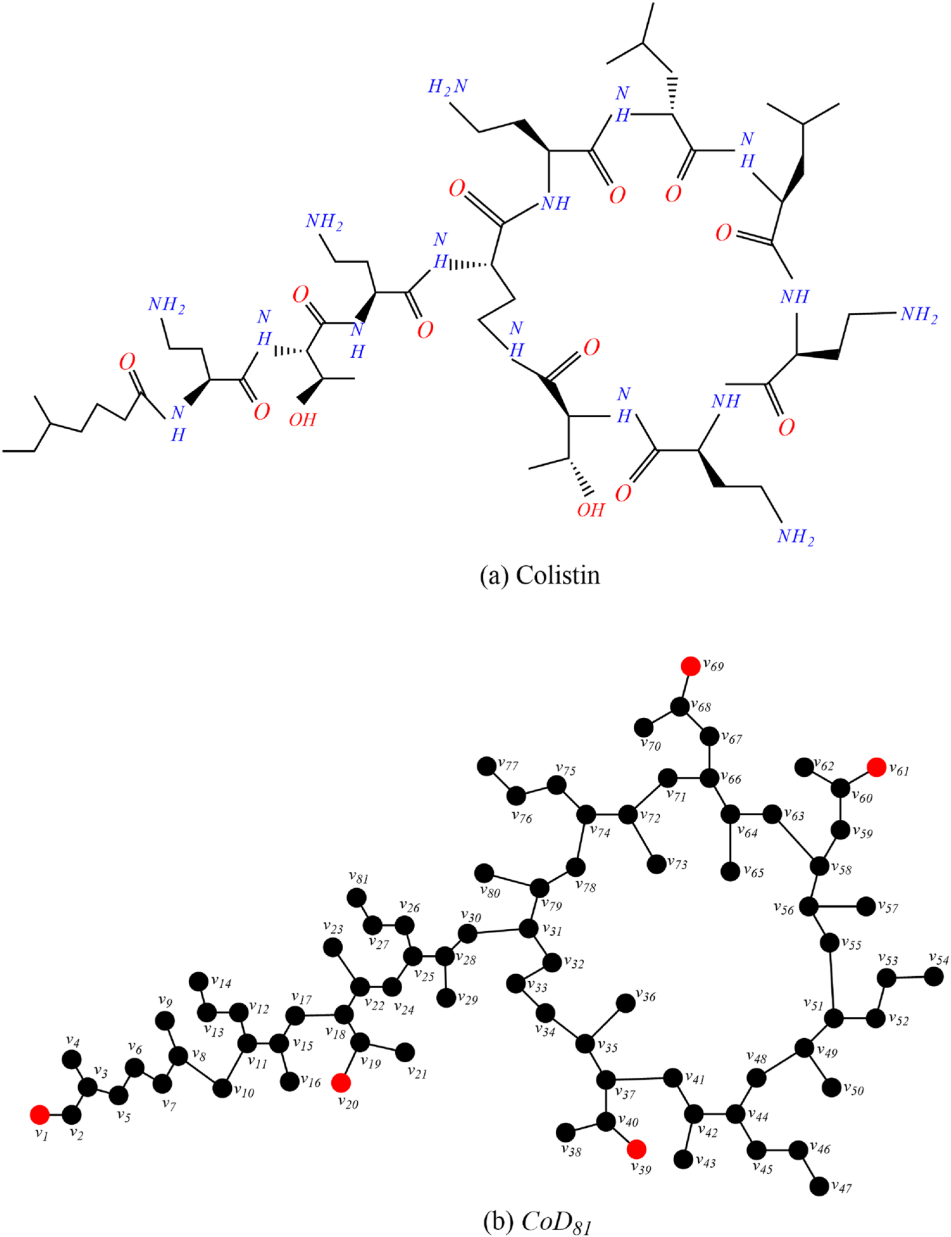
(**a**) Chemical structure of Colistin. (**b**) Labeled hydrogen-suppressed molecular graph $CoD_{81}$ of Colistin.

Colistin^28^ (Polymyxin E), denoted by $CoD_{81}$ is a complex cyclic lipopeptide antibiotic widely used for the treatment of severe infections caused by Gram-negative bacteria. Chemically, it is not a single compound but a mixture of two principal components, namely Colistin A (Polymyxin E$_1$) and Colistin B (Polymyxin E$_2$). The pure base of Colistin A has the molecular formula $C_{53}H_{100}N_{16}O_{13}$, whereas Colistin B possesses the molecular formula $C_{52}H_{98}N_{16}O_{13}$. Owing to its highly intricate cyclic and lipopeptide structure, Colistin exhibits a rich molecular architecture that is well suited for graph-theoretic modeling and structural analysis. The IUPAC name of Colistin A is extremely long and complex due to its cyclic lipopeptide structure; therefore, it is omitted here for readability and provided in the Supplementary Material. The molecular graph representation and the corresponding chemical structure of $CoD_{34}$ are illustrated in Fig. 2a and b, respectively.

### Lemma 3.1

*Let* $CoD_{81}$ *denote the hydrogen-suppressed heavy-atom molecular graph of the drug colistin. Then* $\dim (CoD_{81}) \ge 5$.

### Proof

We aim to demonstrate that $\dim (CoD_{81}) \ge 5$. Assume, for the sake of contradiction, that $\dim (CoD_{81}) \le 4$. Let $S' = \{v_{\alpha }, v_{\beta }, v_{\gamma }, v_{\zeta }\}$ be a resolving set of $CoD_{81}$ consisting of four vertices. Since $CoD_{81}$ contains several symmetric and cyclic substructures, the corresponding neighborhoods of these vertices exhibit substantial overlap. As a consequence, there exist at least two distinct vertices $v_{x}, v_{y} \in N_{8}(v_{\alpha }) \cup N_{8}(v_{\beta }) \cup N_{8}(v_{\gamma }) \cup N_{8}(v_{\zeta })$ whose distance representations with respect to $S'$ are identical; that is,

$$r(v_{x}\mid S') = r(v_{y}\mid S').$$

This contradicts the definition of a resolving set, according to which every pair of distinct vertices must have different metric representations. Hence, the assumption is false, and therefore

$$\dim (CoD_{81}) \ge 5.$$

$$\square$$

### Theorem 3.2

*Let* $CoD_{81}$ *denote the hydrogen-suppressed heavy-atom molecular graph of the drug colistin. Then* $\dim (CoD_{81})=5$.

### Proof

By Lemma 3.1 we have $\dim (CoD_{81})\ge 5$ (the molecular skeleton of colistin contains sufficiently many branching units and symmetric substructures to force a lower bound of 5). We shall exhibit a resolving set of size 5, which will establish equality.

Choose the following five landmarks placed at well-separated terminal and branching loci of the molecule:

$$S=\{v_{1},v_{20},v_{39},v_{61},v_{69}\}.$$

For each vertex $v_{\xi }$ of $CoD_{81}$ define the distance representation with respect to *S* by

$$r(v_{\xi }\mid S)=\bigl (d(v_{\xi },v_{1}),\,d(v_{\xi },v_{20}),\,d(v_{\xi },v_{39}),\,d(v_{\xi },v_{61}),\,d(v_{\xi },v_{69})\bigr ).$$

A direct accumulation of shortest-path lengths along the heavy-atom backbone, carried out block by block, yields the following explicit casewise expressions.

$$\text {for }\xi =1,\ldots ,4:\quad r(v_\xi \mid S)=\Bigl (\xi -1,\ 14-\xi +\alpha _{1}, \ 25-\xi +2\alpha _{1}, \ 30-\xi +2\alpha _{1}, \ 27-\xi +2\alpha _{1} \Bigr ), \text { where }\alpha _{1}=\Bigl \lfloor \frac{\xi -1}{3}\Bigr \rfloor ;$$

$$\text {for }\xi =5,\ldots ,9:\quad r(v_\xi \mid S)=\Bigl (\xi -2,\ 15-\xi +2\alpha _{2}, \ 26-\xi +2\alpha _{2}, \ 31-\xi +2\alpha _{2}, \ 28-\xi +2\alpha _{2} \Bigr ), \text { where }\alpha _{2}\Bigl \lfloor \frac{\xi -5}{3}\Bigr \rfloor ;$$

$$\text {for }\xi = 10,11 :\quad r(v_\xi \mid S)=\Bigl ( \xi -3,\ 16-\xi , \ 27-\xi , \ 32-\xi , \ 29-\xi \Bigr );$$

$$\text {for }\xi = 12,\ldots ,14 :\quad r(v_\xi \mid S)=\Bigl ( \xi -3, \ \xi -6,\ \xi +5 ,\ \xi +10 ,\ \xi +7 \Bigr );$$

$$\text {for }\xi = 15,16:\quad r(v_\xi \mid S)=\Bigl ( \xi -6,\ \xi -11 , \ \xi , \ \xi +5, \ \xi +2 \Bigr );$$

$$\text {for }\xi = 17,18:\quad r(v_\xi \mid S)=\Bigl ( \xi -6,\ 20-\xi , \ 31-\xi , \ 36-\xi , \ 33-\xi \Bigr );$$

$$\text {for }\xi = 19,\ldots , 21:\quad r(v_\xi \mid S)=\Bigl ( 12+\alpha _{3}, \ 20-\xi +3\alpha _{3}, \ 14+\alpha _{3}, \ 19+\alpha _{3}, \ 16+\alpha _{3}\Bigr ),\text { where }\alpha _{3}=\Bigl \lfloor \frac{\xi -18}{2}\Bigr \rfloor ;$$

$$\text {for }\xi = 22,\ldots ,24 :\quad r(v_\xi \mid S)=\Bigl ( 10+\alpha _{4}, \Bigl \lfloor \frac{\xi -15}{2}\Bigr \rfloor , \ 10+\alpha _{4}-2\alpha _{5}, \ 15+\alpha _{4}-2\alpha _{5}, \ 12+\alpha _{4}-2\alpha _{5}\Bigr ),\text { where }\alpha _{4}=\Bigl \lfloor \frac{\xi -17}{2}\Bigr \rfloor \text { and }\alpha _{5}=\Bigl \lfloor \frac{\xi -21}{3}\Bigr \rfloor ;$$

$$\text {for }\xi = 25,\ldots , 27:\quad r(v_\xi \mid S)=\Bigl ( \xi -11,\ \xi -20 , \ \xi -15, \ \xi -10, \ \xi -13 \Bigr );$$

$$\text {for }\xi = 28,29 :\quad r(v_\xi \mid S)=\Bigl ( \Bigl \lfloor \frac{\xi +3}{2}\Bigr \rfloor ,\ \Bigl \lfloor \frac{\xi -15}{2}\Bigr \rfloor ,\ \xi -19 ,\ \xi -14 ,\ \xi -17 \Bigr );$$

$$\text {for }\xi = 30,31 :\quad r(v_\xi \mid S)=\Bigl ( \Bigl \lfloor \frac{\xi +3}{2}\Bigr \rfloor ,\ \Bigl \lfloor \frac{\xi -15}{2}\Bigr \rfloor ,\ 38-\xi ,\ 43-\xi ,\ 40-\xi \Bigr );$$

$$\text {for }\xi = 32,\ldots ,34 :\quad r(v_\xi \mid S)=\Bigl ( \xi -14, \ \xi -23, \ 38-\xi , \ \Bigl \lfloor \frac{\xi -5}{2}\Bigr \rfloor , \ \xi -22\Bigr );$$

$$\text {for }\xi = 35,36 :\quad r(v_\xi \mid S)=\Bigl ( \xi -14, \ \xi -23, \ \xi -32, \ \xi -22, \ \xi -22\Bigr );$$

$$\text {for }\xi = 37,\ldots ,40 :\quad r(v_\xi \mid S)=\Bigl (\xi -15-\alpha _{6}, \ \xi -24-\alpha _{6}, \ 39-\xi +3\alpha _{6}, \ \xi -25-\alpha _{6},\ \xi -23-\alpha _{6} \Bigr ),\text { where }\alpha _{6}=\Bigl \lfloor \frac{\xi -36}{4}\Bigr \rfloor ;$$

$$\text {for }\xi = 41,\ldots , 43:\quad r(v_\xi \mid S)=\Bigl ( \xi -18,\ \xi -27 ,\ \xi -38 , \ 52-\xi +2\alpha _{7},\ 55-\xi +2\alpha _{7} \Bigr ),\text { where }\alpha _{7}=\Bigl \lfloor \frac{\xi -41}{2}\Bigr \rfloor ;$$

$$\text {for }\xi = 44,\ldots , 47:\quad r(v_\xi \mid S)=\Bigl ( \xi -19, \ \xi -28, \ \xi -39, \ \xi -35, \ \xi -32 \Bigr );$$

$$\text {for }\xi = 48,\ldots , 50:\quad r(v_\xi \mid S)=\Bigl ( \xi -22,\ \xi -31 ,\ \xi -42 , \ 56-\xi +2\alpha _{8},\ 59-\xi +2\alpha _{8} \Bigr ),\text { where }\alpha _{8}=\Bigl \lfloor \frac{\xi -48}{2}\Bigr \rfloor ;$$

$$\text {for }\xi = 51,\ldots ,54 :\quad r(v_\xi \mid S)=\Bigl (\xi -23, \ \xi -32, \ \xi -43, \ \xi -45,\ \xi -42 \Bigr );$$

$$\text {for }\xi = 55,\ldots ,57 :\quad r(v_\xi \mid S)=\Bigl ( 83-\xi +2\alpha _{9}, \ 74-\xi +2\alpha _{9},\ \xi -46 ,\ 60-\xi +2\alpha _{9} ,\ 63-\xi +2\alpha _{9} \Bigr ),\text { where }\alpha _{9}=\Bigl \lfloor \frac{\xi -55}{2}\Bigr \rfloor ;$$

$$\text {for }\xi = 58,\ldots ,62 :\quad r(v_\xi \mid S)=\Bigl ( \xi -32,\ \xi -41 -\alpha _{10} ,\ \xi -47 -\alpha _{10} ,\ 61-\xi +3\alpha _{10} ,\ \xi -52 -\alpha _{10} \Bigr ),\text { where }\alpha _{10}=\Bigl \lfloor \frac{\xi -58}{2}\Bigr \rfloor ;$$

$$\text {for }\xi = 63,\ldots , 65:\quad r(v_\xi \mid S)=\Bigl (88-\xi +2\alpha _{11} , \ 79-\xi +2\alpha _{11}, \ \xi -51, \ \xi -59,\ 68-\xi +2\alpha _{11} \Bigr ),\text { where }\alpha _{10}=\Bigl \lfloor \frac{\xi -63}{2}\Bigr \rfloor ;$$

$$\text {for }\xi = 66,\ldots ,70 :\quad r(v_\xi \mid S)=\Bigl ( \xi -43 -\alpha _{12}, \ \xi -52 -\alpha _{12},\ \xi -53 -\alpha _{12} , \ \xi -60 -\alpha _{12},\ 69-\xi +3\alpha _{12} \Bigr ),\text { where }\alpha _{12}=\Bigl \lfloor \frac{\xi -66}{4}\Bigr \rfloor ;$$

$$\text {for }\xi = 71,\ldots ,73 :\quad r(v_\xi \mid S)=\Bigl ( 93-\xi +2\alpha _{13},\ 84-\xi +2\alpha _{13} ,\ 83-\xi +2\alpha _{13} ,\ \xi -64 ,\ \xi -67 \Bigr ),\text { where }\alpha _{13}=\Bigl \lfloor \frac{\xi -71}{2}\Bigr \rfloor ;$$

$$\text {for }\xi = 74,\ldots , 77:\quad r(v_\xi \mid S)=\Bigl (\xi -54,\ \xi -63 ,\ \xi -64 ,\ \xi -65 ,\ \xi -68 \Bigr );$$

$$\begin{aligned} \text {for }\xi = 78,\ldots , 81:\quad r(v_\xi \mid S) & =\Bigl ( 97-\xi +2\alpha _{14}-\alpha _{15},\ 88-\xi +2\alpha _{14}-\alpha _{15} ,\ 87-\xi +2\alpha _{14}+5\alpha _{15} ,\ \xi -68 +5\alpha _{15},\ \xi -71 +5\alpha _{15}\Bigr ), \\ & \quad \quad \text { where }\alpha _{14}=\Bigl \lfloor \frac{\xi -78}{2}\Bigr \rfloor \text { and }\alpha _{15}=\Bigl \lfloor \frac{\xi -78}{3}\Bigr \rfloor .\end{aligned}$$

Each index range above corresponds to a contiguous structural fragment of the colistin heavy-atom graph (terminal sectors, linkers, branching residues, and so on). Within any fixed interval the coordinates of $r(v_\xi \mid S)$ are affine in $\xi$ except at the finitely many breakpoints introduced by the floor terms; moreover, for consecutive indices inside the same interval at least one coordinate changes by exactly $\pm 1$. Hence the map $\xi \mapsto r(v_\xi \mid S)$ is injective on each interval.

At the interfaces between adjacent intervals, traversal across an articulation vertex (a mandatory gateway for shortest paths) produces an additive offset in at least one coordinate of the distance tuple. Therefore no representation that occurs at the start of a new interval can coincide with any representation from the interior of the preceding interval. A direct verification of the end-point and start-point values for each consecutive pair of intervals confirms that there is always at least one differing coordinate.

Thus every vertex of $CoD_{81}$ has a unique distance representation with respect to $S=\{v_{1},v_{20},v_{39},v_{61},v_{69}\}$, so *S* is a resolving set. Combining this with the lemma that $\dim (CoD_{81})\ge 5$ yields $\dim (CoD_{81})=5$, completing the proof. $\square$

## Metric dimension of berberine

**Fig. 3:**
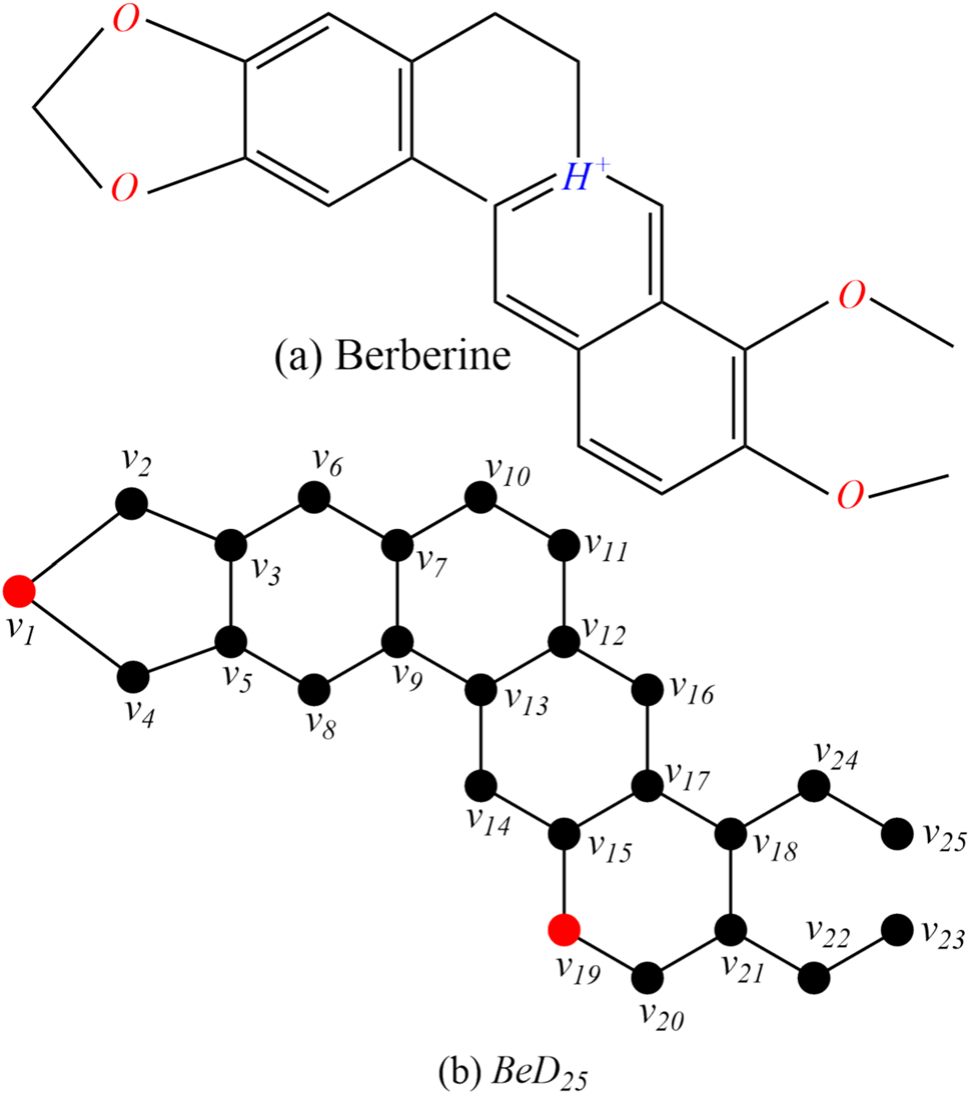
(**a**) Chemical structure of berberine. (**b**) Labeled hydrogen-suppressed molecular graph $BeD_{25}$ of berberine.

Berberine^29^, denoted by $BeD_{25}$, is a naturally occurring isoquinoline alkaloid widely recognized for its broad-spectrum antimicrobial and anti-biofilm properties. It has the molecular formula $C_{20}H_{18}N_{4}^{+}$. Owing to its rigid and planar molecular framework, Berberine serves as an appropriate candidate for graph-theoretic investigation and metric dimension analysis. The IUPAC name of Berberine is 9,10-dimethoxy-7,8,13,13a-tetradehydro-2$^\prime H$-[1,3]dioxolo[4$^\prime$,5$^\prime$:2,3]berbin-7-ium. The molecular graph representation and the corresponding chemical structure of $BeD_{25}$ are illustrated in Fig. 3a and b, respectively.

### Theorem 4.1

*Let* $BeD_{25}$ *denote the molecular graph of the drug berberine. Then* $\dim (BeD_{25})=2$.

### Proof

We regard $BeD_{25}$ as the hydrogen-suppressed heavy-atom graph of berberine: vertices correspond to non-hydrogen (heavy) atoms and edges correspond to covalent bonds between them. Because $BeD_{25}$ contains cycles and is not a path, we have $\dim (BeD_{25})\ge 2$ (indeed, the only connected graphs with metric dimension 1 are paths).

We now construct a resolving set of size 2. Choose the two landmarks on opposite terminal portions of the molecule, labelled here as $v_{1}$ (on the left terminal ring) and $v_{19}$ (on the right terminal region); set $S=\{v_{1},v_{19}\}$. For each vertex $v_\xi$ ($1\le \xi \le n$) define the distance representation with respect to *S* by

$$r(v_\xi \mid S)=\bigl (d(v_\xi ,v_1),\,d(v_\xi ,v_{19})\bigr ).$$

(For the berberine heavy-atom graph studied here the vertex set is $\{v_1,\dots ,v_{25}\}$, so $n=25$.)

By following shortest paths through the molecular backbone from the left terminal region toward the right terminal region, one observes the same qualitative opposition of trends that appeared in the previous example: as one moves from left to right along contiguous structural fragments, $d(\,\cdot ,v_1)$ is (piecewise) nondecreasing while $d(\,\cdot ,v_{19})$ is (piecewise) nonincreasing, with discrete jumps when crossing articulation points (ring $\leftrightarrow$ linker or linker $\leftrightarrow$ chain). A direct computation of shortest-path lengths block by block yields the following explicit casewise expressions for the distance pairs.

$$\text {for }\xi =1,\ldots ,6:\quad r(v_\xi \mid S)=\Bigl ( \xi -1-2\Bigl \lfloor \frac{\xi -1}{3}\Bigr \rfloor ,\ 8-\Bigl \lfloor \frac{\xi -1}{2}\Bigr \rfloor \Bigr );$$

$$\text {for }\xi =7,\ldots ,9:\quad r(v_\xi \mid S)=\Bigl (11-\xi +2\Bigl \lfloor \frac{\xi -7}{2}\Bigr \rfloor ,\ 5-\Bigl \lfloor \frac{\xi -7}{2}\Bigr \rfloor \Bigr );$$

$$\text {for }\xi =10,\ldots ,12:\quad r(v_\xi \mid S)=\Bigl ( \Bigl \lfloor \frac{\xi +1}{2}\Bigr \rfloor ,\ 16-\xi \Bigr );$$

$$\text {for }\xi =13,\ldots ,15:\quad r(v_\xi \mid S)=\Bigl (\xi -8 ,\ 16-\xi \Bigr );$$

$$\text {for }\xi =16,\ldots ,18:\quad r(v_\xi \mid S)=\Bigl ( \xi -9,\ 19-\xi +2\Bigl \lfloor \frac{\xi -16}{2}\Bigr \rfloor \Bigr );$$

$$\text {for }\xi =19,\ldots ,23:\quad r(v_\xi \mid S)=\Bigl (\xi -11 ,\ \xi -19 \Bigr );$$

$$\text {for }\xi =24,25:\quad r(v_\xi \mid S)=\Bigl (\xi -14 ,\ \xi -20 \Bigr ).$$

Each index interval above corresponds to a contiguous structural fragment of the berberine heavy-atom graph (left ring sector; left linker; central fused ring/chain region; right linker; right ring sector, etc.). Within any single interval both coordinates of $r(v_\xi \mid S)$ are affine (linear in $\xi$) except at finitely many breakpoints introduced by the floor terms. Moreover, between successive indices $\xi$ and $\xi +1$ inside the same interval at least one of the two coordinates changes by $\pm 1$. Hence, within each interval the map $\xi \mapsto r(v_\xi \mid S)$ is injective.

At the boundaries between intervals an articulation (a gateway vertex through which every shortest path must pass when crossing from one block to the next) forces an additional increment in one of the distance coordinates relative to the last interior value of the previous interval. Consequently, a vector produced at the start of a new interval cannot coincide with any vector from the interior of the preceding interval. A routine check of the displayed endpoint and initial values for consecutive intervals confirms that the terminal pair in each interval differs from the initial pair in the next interval in at least one coordinate.

Therefore all vertices $v_\xi$ ($1\le \xi \le 25$) have distinct distance representations with respect to $S=\{v_1,v_{19}\}$, so *S* is a resolving set. Combining this with the earlier observation that $\dim (BeD_{25})\ge 2$ yields $\dim (BeD_{25})=2$, as required. $\square$

## Metric dimension of usnic acid

**Fig. 4:**
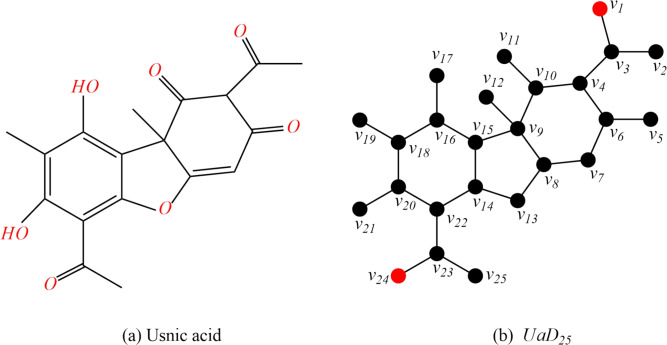
(**a**) Chemical structure of usnic acid. (**b**) Labeled hydrogen-suppressed molecular graph $UaD_{25}$ of usnic acid.

Usnic acid^30^, denoted by $UaD_{25}$, is a naturally occurring secondary metabolite commonly isolated from lichens and is well known for its antimicrobial and anti-biofilm activities. It has the molecular formula $C_{18}H_{16}O_{7}$. Owing to its polycyclic and rigid molecular framework, Usnic acid provides a suitable structure for graph-theoretic modeling and metric dimension analysis. The IUPAC name of Usnic acid is 2,6-diacetyl-7,9-dihydroxy-8,9*b*-dimethyldibenzo[*b*, *d*]furan-1,3(2*H*, 9*bH*)-dione. The molecular graph representation and the corresponding chemical structure of $UaD_{25}$ are illustrated in Fig. 4a and b, respectively.

### Theorem 5.1

*Let* $UaD_{25}$ *denote the molecular graph of the drug usnic acid. Then* $\dim (UaD_{25})=2$.

### Proof

We consider $UaD_{25}$ as the hydrogen-suppressed heavy-atom graph of usnic acid, where each vertex represents a non-hydrogen atom and each edge corresponds to a covalent bond. Since the graph $UaD_{25}$ contains several cycles and is not a simple path, it follows that $\dim (UaD_{25}) \ge 2$, as only paths possess metric dimension 1.

To demonstrate that $\dim (UaD_{25})=2$, we exhibit a resolving set of size 2. Select two vertices located at opposite terminal regions of the molecular skeleton, labelled as $v_{1}$ (on the left terminal group) and $v_{24}$ (on the right terminal group). Define

$$S=\{v_{1},v_{24}\}, \quad r(v_\xi \mid S)=\bigl (d(v_\xi ,v_{1}),\,d(v_\xi ,v_{24})\bigr ),$$

for $1\le \xi \le n$, where $n=25$ represents the number of vertices in $UaD_{25}$.

Following the molecular backbone from one end to the other, $d(\cdot ,v_{1})$ tends to increase, while $d(\cdot ,v_{24})$ generally decreases, with changes in slope at articulation points (ring $\leftrightarrow$ chain, chain $\leftrightarrow$ substituent). The resulting pairwise distances for all vertices can be expressed casewise as follows:

$$\text {for }\xi =1,2:\quad r(v_\xi \mid S)=\Bigl ( 2\Bigl \lfloor \frac{\xi }{2}\Bigr \rfloor ,\ 9 \Bigr );$$

$$\text {for }\xi =3,\ldots ,5:\quad r(v_\xi \mid S)=\Bigl ( \xi -2+\Bigl \lfloor \frac{\xi -3}{2}\Bigr \rfloor ,\ 11-\xi +2\Bigl \lfloor \frac{\xi -3}{2}\Bigr \rfloor \Bigr );$$

$$\text {for }\xi =6,\ldots ,8:\quad r(v_\xi \mid S)=\Bigl (\xi -3 ,\ 13-\xi \Bigr );$$

$$\text {for }\xi =9,\ldots ,11:\quad r(v_\xi \mid S)=\Bigl ( 13-\xi +2\Bigl \lfloor \frac{\xi -9}{2}\Bigr \rfloor ,\ \xi -4\Bigr );$$

$$\text {for }\xi =12,\ldots ,14:\quad r(v_\xi \mid S)=\Bigl ( \xi -7-\Bigl \lfloor \frac{\xi -12}{2}\Bigr \rfloor ,\ 18-\xi -\Bigl \lfloor \frac{\xi -11}{2}\Bigr \rfloor \Bigr );$$

$$\text {for }\xi =15,\ldots ,17:\quad r(v_\xi \mid S)=\Bigl ( \xi -10,\ \xi -11 \Bigr );$$

$$\text {for }\xi =18,\ldots ,21:\quad r(v_\xi \mid S)=\Bigl ( \Bigl \lfloor \frac{\xi -3}{2}\Bigr \rfloor ,\ \xi -14-3\Bigl \lfloor \frac{\xi -18}{2}\Bigr \rfloor \Bigr );$$

$$\text {for }\xi =22,\ldots ,25:\quad r(v_\xi \mid S)=\Bigl ( \xi -15-\Bigl \lfloor \frac{\xi -22}{3}\Bigr \rfloor ,\ 24-\xi +2\Bigl \lfloor \frac{\xi -22}{3}\Bigr \rfloor \Bigr ).$$

Each of these intervals corresponds to a specific fragment of the molecular skeleton (rings, linkers, or aliphatic chains). Within any single interval, both coordinates of $r(v_\xi \mid S)$ vary linearly with $\xi$, except for discrete shifts introduced by the floor terms. Moreover, between consecutive vertices $\xi$ and $\xi +1$, at least one coordinate changes by $\pm 1$, ensuring that the mapping $\xi \mapsto r(v_\xi \mid S)$ is injective within each block.

At the interfaces between blocks, crossing an articulation point necessarily alters one of the distances by at least 1, producing an offset that prevents any overlap of distance pairs between adjacent intervals. A direct comparison of the terminal and initial pairs in consecutive ranges confirms that they differ in at least one coordinate.

Consequently, all vertices in $UaD_{25}$ have unique distance representations with respect to $S=\{v_{1},v_{24}\}$, showing that *S* is a resolving set. Hence, $\dim (UaD_{25})=2$, which completes the proof. $\square$

## Metric dimension of ellagic acid

**Fig. 5:**
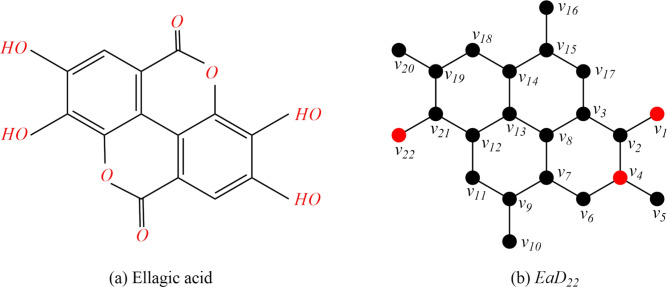
(**a**) Chemical structure of ellagic acid. (**b**) Labeled hydrogen-suppressed molecular graph $EaD_{22}$ of ellagic acid.

Ellagic Acid^31^, represented as $EaD_{22}$, is a naturally existing polyphenolic compound that can be found in a variety of fruits, vegetables, and medicinal herbs for its antimicrobial and anti-biofilm properties. The chemical structure of Ellagic Acid consists of the molecular formula $\mathrm {C_{14}H_{6}O_{8}}$. The polycyclic nature of Ellagic Acid, combined with the various functional groups, makes it a suitable candidate for modeling studies involving graph theory. The IUPAC name of Ellagic acid is 2,3,7,8-tetrahydroxy[1]benzopyrano[5,4,3-*cde*][1]benzopyran-5,10-dione. The molecular graph representation and the corresponding chemical structure of $EaD_{22}$ are illustrated in Fig. 5a and b, respectively.

### Lemma 6.1

*Let* $EaD_{22}$ *denote the hydrogen-suppressed molecular graph of the drug ellagic acid. Then* $\dim (EaD_{22})\ge 3$.

### Proof

We intend to establish that $\dim (EaD_{22}) \ge 3$. Suppose, for the sake of contradiction, that $\dim (EaD_{22}) \le 2$. Then there exists a resolving set $S' = \{v_{\alpha }, v_{\beta }\}$ for $EaD_{22}$ consisting of two vertices. The molecular graph of $EaD_{22}$ possesses a high degree of symmetry together with several cyclic substructures, which gives rise to repeated distance patterns among its vertices. Consequently, there exist at least two distinct vertices $v_{x}, v_{y} \in N_{3}(v_{\alpha }) \cup N_{3}(v_{\beta })$ such that

$$r(v_{x}\mid S') = r(v_{y}\mid S').$$

This contradicts the defining property of a resolving set, namely that distinct vertices must have distinct metric representations with respect to it. Therefore, the assumption is invalid, and we conclude that

$$\dim (EaD_{22}) \ge 3.$$

$$\square$$

### Theorem 6.2

*Let* $EaD_{22}$ *denote the hydrogen-suppressed molecular graph of the drug ellagic acid. Then the metric dimension of* $EaD_{22}$ *is 3*.

### Proof

By structural inspection, $EaD_{22}$ consists of two fused aromatic rings with several oxygen-containing substituents. Since this molecular graph contains multiple cycles with symmetric branches, it follows from Lemma 6.1 that $\dim (EaD_{22}) \ge 3$.

We now show that three appropriately chosen vertices can resolve all others in $EaD_{22}$. Let $S=\{v_{1},v_{4},v_{22}\}$, where $v_{1}$ lies on the left terminal ring, $v_{4}$ on the central junction, and $v_{22}$ on the right periphery of the molecule. For each vertex $v_{\xi }$, the distance representation with respect to *S* is defined as

$$r(v_{\xi }\mid S)=\bigl (d(v_{\xi },v_{1}),\,d(v_{\xi },v_{4}),\,d(v_{\xi },v_{22})\bigr ).$$

A systematic traversal of the molecular framework, recording the shortest-path distances to these three landmarks, yields the following coordinate expressions:

$$\text {for }\xi = 1,\ldots ,3 :\quad r(v_\xi \mid S)=\Bigl (\xi -1 ,\ 3-\xi +2\Bigl \lfloor \frac{\xi -1}{2}\Bigr \rfloor ,\ 8-\xi \Bigr );$$

$$\text {for }\xi = 4,\ldots ,7 :\quad r(v_\xi \mid S)=\Bigl ( \Bigl \lfloor \frac{\xi +1}{2}\Bigr \rfloor ,\ \Bigl \lfloor \frac{\xi -3}{2}\Bigr \rfloor ,\ \xi +3-3\Bigl \lfloor \frac{\xi -4}{2}\Bigr \rfloor -2\Bigl \lfloor \frac{\xi -4}{3}\Bigr \rfloor \Bigr );$$

$$\text {for }\xi = 8,\ldots ,10 :\quad r(v_\xi \mid S)=\Bigl (\xi -5+\Bigl \lfloor \frac{\xi -7}{2}\Bigr \rfloor ,\ \Bigl \lfloor \frac{\xi -2}{2}\Bigr \rfloor ,\ \Bigl \lfloor \frac{\xi }{2}\Bigr \rfloor \Bigr );$$

$$\text {for }\xi = 11,\ldots ,13 :\quad r(v_\xi \mid S)=\Bigl ( 17-\xi ,\ \xi -7-2\Bigl \lfloor \frac{\xi -11}{2}\Bigr \rfloor ,\ 14-\xi +2\Bigl \lfloor \frac{\xi -11}{2}\Bigr \rfloor \Bigr );$$

$$\text {for }\xi = 14,\ldots ,16 :\quad r(v_\xi \mid S)=\Bigl ( 19-\xi +2\Bigl \lfloor \frac{\xi -14}{2}\Bigr \rfloor ,\ 19-\xi +2\Bigl \lfloor \frac{\xi -14}{2}\Bigr \rfloor ,\ \xi -10 \Bigr );$$

$$\text {for }\xi = 17,\ldots ,19 :\quad r(v_\xi \mid S)=\Bigl ( \xi -14+2\Bigl \lfloor \frac{\xi -16}{2}\Bigr \rfloor ,\ \xi -14+2\Bigl \lfloor \frac{\xi -16}{2}\Bigr \rfloor ,\ 23-\xi -2\Bigl \lfloor \frac{\xi -16}{2}\Bigr \rfloor \Bigr );$$

$$\text {for }\xi = 20,\ldots , 22:\quad r(v_\xi \mid S)=\Bigl ( \xi -12-3\Bigl \lfloor \frac{\xi -19}{2}\Bigr \rfloor ,\ \xi -12-3\Bigl \lfloor \frac{\xi -19}{2}\Bigr \rfloor ,\ 23-\xi -\Bigl \lfloor \frac{\xi -19}{2}\Bigr \rfloor \Bigr ).$$

Every one of the above index values is associated with a different structural part of the molecule, ranging from the left aromatic ring to the bridge area and finally to the right ring system. For each index value, the coordinates of $r(v_{\xi }|S)$ change monotonously or piecewise linearly with respect to $\xi$, so that each pair of successive vertices has a different distance triple.

A ring junction or a bond connection crossing through two adjacent intervals changes the value of at least one distance coordinate. It follows that no vertex in any structure block can have the same distance representation as any other vertex in another structure block.

Consequently, the mapping $\xi \mapsto r(v_{\xi }\mid S)$ is injective over $V(EaD_{22})$, confirming that the selected set $S=\{v_{1},v_{4},v_{22}\}$ resolves the graph completely. By the lemma establishing $\dim (EaD_{22}) \ge 3$, it follows that

$$\dim (EaD_{22}) = 3.$$

$$\square$$

## Metric dimension of curcumin

**Fig. 6:**
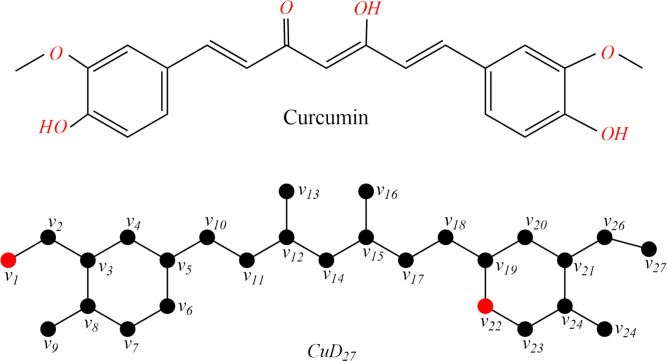
(**a**) Chemical structure of curcumin. (**b**) Labeled hydrogen-suppressed molecular graph $CuD_{27}$ of curcumin.

Curcumin^32^, denoted by $CuD_{27}$, is a naturally occurring polyphenolic compound widely known for its antioxidant, antimicrobial, and anti-biofilm properties. It has the molecular formula $C_{21}H_{20}O_{6}$. Owing to its conjugated structure and flexible molecular backbone, Curcumin provides a suitable framework for graph-theoretic modeling and metric dimension analysis. The IUPAC name of Curcumin is (1*E*, 6*E*)-1,7-bis(4-hydroxy-3-methoxyphenyl)hepta-1,6-diene-3,5-dione. The molecular graph representation and the corresponding chemical structure of $CuD_{27}$ are illustrated in Fig. 6a and b, respectively.

### Theorem 7.1

*Let* $CuD_{27}$ *denote the hydrogen-suppressed heavy-atom molecular graph of curcumin. Then* $\dim (CuD_{27})=2$.

### Proof

We view $CuD_{27}$ as the heavy-atom graph of curcumin: vertices represent non-hydrogen atoms and edges represent covalent bonds. Because $CuD_{27}$ contains cycles and is not a path, we have $\dim (CuD_{27})\ge 2$ (indeed, the only connected graphs of metric dimension 1 are paths).

We now produce a resolving set of size two. Choose landmarks situated on opposite terminal portions of the molecular skeleton, labelled here as $v_{1}$ (left terminal) and $v_{22}$ (right terminal), and set $S=\{v_{1},v_{22}\}$.

For each vertex $v_{\xi }$ ($1\le \xi \le n$; in the present labelling $n=27$) define the distance representation

$$r(v_\xi \mid S)=\bigl (d(v_\xi ,v_{1}),\,d(v_\xi ,v_{22})\bigr ).$$

As one traverses the heavy-atom backbone from the left terminal region toward the right terminal region, any shortest path to $v_{1}$ is (piecewise) nonincreasing in proximity to the left end while any shortest path to $v_{22}$ is (piecewise) nondecreasing; equivalently, $d(\cdot ,v_{1})$ is (piecewise) nondecreasing and $d(\cdot ,v_{22})$ is (piecewise) nonincreasing as one moves left-to-right along contiguous structural fragments. Discrete increments occur when crossing articulation points (ring $\leftrightarrow$ linker or linker $\leftrightarrow$ chain). This opposing behaviour of the two coordinates is the mechanism that yields unique ordered distance pairs.

A straightforward accumulation of shortest-path lengths block-by-block produces the following explicit casewise formulas for the metric representations.

$$\text {for }\xi = 1,\ldots ,5 :\quad r(v_\xi \mid S)=\Bigl ( \xi -1,\ 14-\xi \Bigr );$$

$$\text {for }\xi = 6,\ldots , 9:\quad r(v_\xi \mid S)=\Bigl ( 11-\xi ,\ \xi +5 \Bigr );$$

$$\text {for }\xi = 10,\ldots , 13:\quad r(v_\xi \mid S)=\Bigl ( \xi -5,\ 18-\xi +2\Bigl \lfloor \frac{\xi -10}{3}\Bigr \rfloor \Bigr );$$

$$\text {for }\xi = 14,\ldots ,16 :\quad r(v_\xi \mid S)=\Bigl ( \xi -6,\ 19-\xi +2\Bigl \lfloor \frac{\xi -14}{2}\Bigr \rfloor \Bigr );$$

$$\text {for }\xi = 17,\ldots , 19:\quad r(v_\xi \mid S)=\Bigl ( \xi -7,\ 20-\xi \Bigr );$$

$$\text {for }\xi = 20,21 :\quad r(v_\xi \mid S)=\Bigl (\xi -7 ,\ \xi -18 \Bigr );$$

$$\text {for }\xi = 22,\ldots , 25:\quad r(v_\xi \mid S)=\Bigl (\xi -9 ,\ \xi -22 \Bigr );$$

$$\text {for }\xi = 26,27 :\quad r(v_\xi \mid S)=\Bigl ( \xi -11,\ \xi -22 \Bigr ).$$

Each index range above corresponds to a contiguous structural fragment of the curcumin heavy-atom graph (terminal ring sectors, linkers, central chain, and so forth). Within any single range both coordinates of $r(v_\xi \mid S)$ are affine functions of $\xi$, except at finitely many breakpoints introduced by the floor terms. Moreover, for two consecutive indices $\xi$ and $\xi +1$ inside the same range at least one of the two coordinates changes by $\pm 1$. Hence the map $\xi \mapsto r(v_\xi \mid S)$ is injective on each interval.

At boundaries between adjacent index intervals an articulation (a gateway vertex through which every shortest path must pass when crossing between blocks) produces an offset: crossing the articulation increases at least one of the two distance coordinates by 1 (relative to the last interior value of the preceding block). Therefore a pair arising at the start of a new interval cannot coincide with any pair from the interior of the previous interval. A direct check of the terminal and initial pairs for consecutive intervals confirms that each terminal pair differs from the next initial pair in at least one coordinate.

Consequently all vertices $v_{\xi }$ ($1\le \xi \le 27$) have distinct metric representations with respect to $S=\{v_{1},v_{22}\}$; thus *S* is a resolving set. Combining this with the earlier observation that $\dim (CuD_{27})\ge 2$ yields $\dim (CuD_{27})=2$, as required. $\square$

## Metric dimension of epigallocatechin gallate

**Fig. 7:**
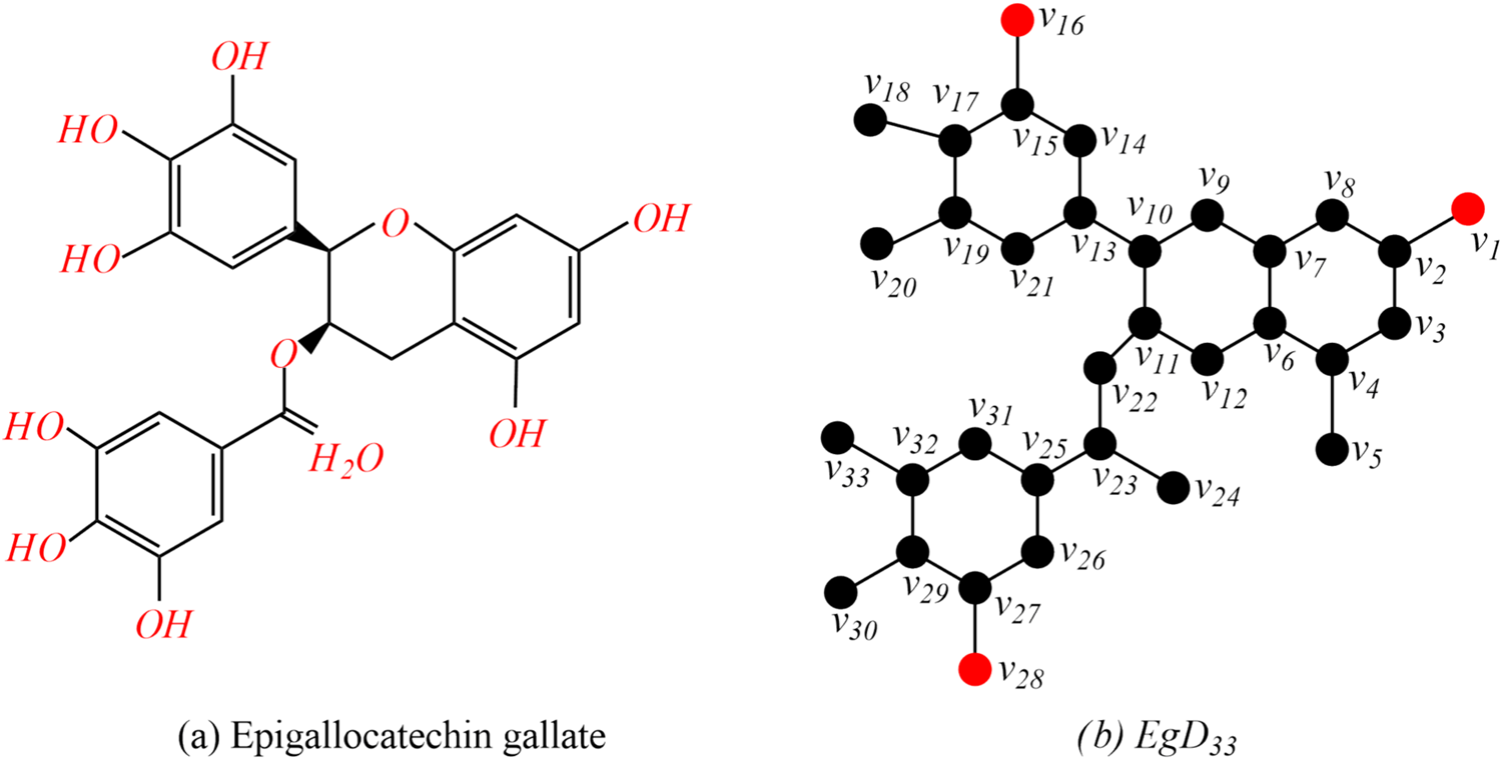
(**a**) Chemical structure of epigallocatechin gallate. (**b**) Labeled hydrogen-suppressed molecular graph $EgD_{33}$ of epigallocatechin gallate.

Epigallocatechin gallate^33^, denoted by $EgD_{33}$, is a naturally occurring polyphenolic compound most commonly found in green tea and is well known for its antioxidant, antimicrobial, and anti-biofilm activities. It has the molecular formula $C_{22}H_{18}O_{11}$. Owing to its polycyclic structure and the presence of multiple hydroxyl functional groups, Epigallocatechin gallate provides a suitable molecular framework for graph-theoretic modeling and metric dimension analysis. The IUPAC name of Epigallocatechin gallate is (2*R*, 3*R*)-3$^\prime$,4$^\prime$,5,5$^\prime$,7-pentahydroxyflavan-3-yl 3,4,5-trihydroxybenzoate. The molecular graph representation and the corresponding chemical structure of $EgD_{33}$ are illustrated in Fig. 7a and b, respectively.

### Lemma 8.1

*Let* $EgD_{33}$ *denote the hydrogen-suppressed heavy-atom molecular graph of the drug Epigallocatechin gallate. Then *$\dim (EaD_{22})\ge 3$.

### Proof

We aim to prove that $\dim (EgD_{33}) \ge 3$. Assume, to the contrary, that $\dim (EgD_{33}) \le 2$. Let $S' = \{v_{\alpha }, v_{\beta }\}$ be a resolving set of $EgD_{33}$ consisting of two vertices.

First, consider the case in which the vertices of $S'$ are not leaves of $EgD_{33}$. The molecular graph $EgD_{33}$ contains several symmetric and cyclic substructures, which create strong similarities among the corresponding neighborhoods. Therefore, there exist at least two distinct vertices $v_{x}, v_{y} \in N_{3}(v_{\alpha }) \cup N_{3}(v_{\beta })$ whose distance representations with respect to $S'$ coincide; that is,

$$r(v_{x}\mid S') = r(v_{y}\mid S').$$

Next, consider the case in which the vertices of $S'$ are leaves of $EgD_{33}$. Owing to the extended cyclic connectivity and the symmetric placement of the leaf attachments, an analogous phenomenon occurs in larger neighborhoods. Hence, there exist at least two distinct vertices $v_{x}, v_{y} \in N_{10}(v_{\alpha }) \cup N_{10}(v_{\beta })$ such that

$$r(v_{x}\mid S') = r(v_{y}\mid S').$$

In both cases, we obtain a contradiction to the definition of a resolving set, since distinct vertices must have distinct metric representations with respect to $S'$. Therefore, the initial assumption is false, and it follows that

$$\dim (EgD_{33}) \ge 3.$$

$$\square$$

### Theorem 8.2

*Let* $EgD_{33}$ *denote the hydrogen-suppressed heavy-atom molecular graph of the drug Epigallocatechin gallate. Then* $\dim (EgD_{33})=3$.

### Proof

By Lemma 8.1, since $EgD_{33}$ possesses multiple branching and ring structures with interlinked aromatic and aliphatic segments, it follows that $\dim (EgD_{33}) \ge 3$.

We now prove that three vertices are sufficient to resolve the entire graph. Take the set $S=\{v_{1},v_{16},v_{28}\}$, where the respective landmarks are represented by the vertices on the left terminal aromatic ring, the central ring, and the right terminal region of the EGCG graph.

For each vertex $v_{\xi }$, $1 \le \xi \le n$, the distance representation with respect to *S* is given by

$$r(v_{\xi }\mid S)=\bigl (d(v_{\xi },v_{1}),\, d(v_{\xi },v_{16}),\, d(v_{\xi },v_{28})\bigr ).$$

A careful traversal of the molecular skeleton, accumulating the shortest-path distances to these three landmarks, yields the following explicit casewise formulas.

$$\text {for }\xi = 1,\ldots ,5 :\quad r(v_\xi \mid S)=\Bigl ( \xi -1,\ 10-\xi +2\Bigl \lfloor \frac{\xi -1}{2}\Bigr \rfloor ,\ 13-\xi +2\Bigl \lfloor \frac{\xi -1}{4}\Bigr \rfloor \Bigr );$$

$$\text {for }\xi = 6,\ldots , 8:\quad r(v_\xi \mid S)=\Bigl ( 10-\xi ,\ 13-\xi +2\Bigl \lfloor \frac{\xi -6}{2}\Bigr \rfloor ,\ \xi +2\Bigr );$$

$$\text {for }\xi = 9,\ldots ,11 :\quad r(v_\xi \mid S)=\Bigl ( \xi -5,\ 14-\xi +2\Bigl \lfloor \frac{\xi -9}{2}\Bigr \rfloor ,\ 17-\xi \Bigr );$$

$$\text {for }\xi = 12,\ldots ,16 :\quad r(v_\xi \mid S)=\Bigl ( \xi -7,\ 18-\xi -2\Bigl \lfloor \frac{\xi -9}{4}\Bigr \rfloor ,\ \xi -5 \Bigr );$$

$$\text {for }\xi = 17,\ldots , 21:\quad r(v_\xi \mid S)=\Bigl ( \xi -8-3\Bigl \lfloor \frac{\xi -17}{2}\Bigr \rfloor ,\ \Bigl \lfloor \frac{\xi -12}{2}\Bigr \rfloor ,\ \xi -6-3\Bigl \lfloor \frac{\xi -17}{2}\Bigr \rfloor \Bigr );$$

$$\text {for }\xi = 22,\ldots , 26:\quad r(v_\xi \mid S)=\Bigl ( \xi -15,\ \xi -16-\Bigl \lfloor \frac{\xi -22}{3}\Bigr \rfloor ,\ 27-\xi \Bigr );$$

$$\text {for }\xi = 27,28 :\quad r(v_\xi \mid S)=\Bigl ( \xi -16,\ \Bigl \lfloor \frac{\xi -6}{2}\Bigr \rfloor ,\ 28-\xi \Bigr );$$

$$\text {for }\xi = 29,30 :\quad r(v_\xi \mid S)=\Bigl ( \xi -16,\ \Bigl \lfloor \frac{\xi -6}{2}\Bigr \rfloor ,\ \xi -27\Bigr );$$

$$\text {for }\xi = 31,\ldots , 33:\quad r(v_\xi \mid S)=\Bigl ( \xi -21,\ \xi -22 ,\ 35-\xi +2\Bigl \lfloor \frac{\xi -31}{2}\Bigr \rfloor \Bigr ).$$

Each index range corresponds to a distinct structural fragment of the EGCG molecule (for example, aromatic ring systems, linker groups, or hydroxylated branches). Within any given interval, each coordinate of $r(v_\xi \mid S)$ varies in a piecewise-linear fashion with respect to $\xi$, except at finitely many discontinuities determined by the floor terms. For any consecutive vertices $\xi$ and $\xi +1$ in the same interval, at least one coordinate changes by $\pm 1$, ensuring injectivity of the mapping $\xi \mapsto r(v_\xi \mid S)$.

At the junctions between consecutive intervals, transitions through articulation vertices (such as ring-to-linker or linker-to-branch connections) induce additive offsets in at least one distance coordinate. Consequently, no vertex in a new interval can share the same representation as any vertex in a preceding one. A verification of the boundary values between successive cases confirms that all representations are pairwise distinct.

Thus, the set $S=\{v_{1},v_{16},v_{28}\}$ uniquely resolves all vertices of $EgD_{33}$. Combined with the lemma ensuring $\dim (EgD_{33})\ge 3$, we conclude that

$$\dim (EgD_{33}) = 3.$$

$$\square$$

### Chemical implications of metric dimension in anti-biofilm molecular graphs

In chemical graph theory, the metric dimension of a molecular graph is the minimum number of landmark vertices required to distinguish all heavy atoms through their distance coordinates. From this viewpoint, it quantifies the extent to which the topology of a molecule can be encoded by a small set of reference positions. A lower metric dimension indicates that the graph can be resolved efficiently from a few landmarks, whereas a higher metric dimension reflects the presence of stronger topological repetition, branching, cyclic overlap, or partial symmetry, all of which create greater ambiguity in distance-based identification.

For the anti-biofilm compounds considered in this work, the computed values provide a comparative description of their structural resolvability. In particular, chlorhexidine, berberine, usnic acid, and curcumin have metric dimension 2, which shows that two suitably chosen vertices are sufficient to distinguish all vertices in their hydrogen-suppressed molecular graphs. By contrast, ellagic acid and epigallocatechin gallate require three landmarks, while colistin requires five. This indicates that the latter graphs possess a more intricate distance structure, caused by a richer combination of cyclic organization, branching, and repeated local environments.

At the same time, it is important to interpret these results carefully. Metric dimension is not a complete descriptor of molecular structure, nor does it uniquely determine the full chemical architecture of a compound. Distinct molecules may share the same metric dimension while differing significantly in ring composition, functional groups, or global geometry. Thus, the fact that two molecular graphs have the same metric dimension does not imply that they are chemically similar. Rather, it means that they require the same minimum number of landmarks for distance-based vertex identification. In this sense, metric dimension should be regarded as a coarse but informative structural descriptor that captures one specific aspect of molecular organization, namely the degree of distinguishability of atomic positions in the underlying graph.

This interpretation is chemically meaningful because atoms that are difficult to distinguish by graph distances often occur in regions with repeated motifs, comparable substituent environments, or partial structural symmetry. Hence, metric dimension can serve as a complementary topological invariant for graph-based molecular characterization, canonical indexing, and descriptor construction in QSPR/QSAR studies. However, it should not be viewed as a direct predictor of anti-biofilm activity. Biological activity depends on a much broader range of factors, including stereochemistry, electronic effects, intermolecular interactions, and physicochemical properties that are not encoded by metric dimension alone.

We also emphasize that the present study is based on a selected family of seven anti-biofilm compounds and is intended as a mathematically rigorous investigation of representative examples rather than a complete statistical analysis across chemical space. Since the determination of metric dimension is computationally difficult in general, large-scale studies require substantial algorithmic and computational effort. A broader investigation involving larger datasets, additional chemical classes, and computational heuristics for estimating metric dimension is part of our ongoing work and will be presented separately.

One aspect that deserves special emphasis is that metric dimension is not a complete structural invariant. Indeed, it is possible for distinct chemical structures to have the same metric dimension. This is because this measure only considers the minimum number of landmarks required for distance-based vertex identification. Therefore, the distinction between berberine and ellagic acid structures is not based only on the number of their common cycle structures. Rather, it is based on the degree of repetition, symmetry, and structural similarity of their molecular graphs. In particular, it is possible for berberine to be identified using only two landmarks, while ellagic acid requires a third landmark because of the high degree of symmetry and repetition present in its hydrogen-suppressed graph.

## Conclusion

In this paper, we determined the metric dimension of the hydrogen-suppressed molecular graphs of several important anti-biofilm compounds. The obtained results show that metric dimension provides a rigorous distance-based invariant for comparing these graphs from the perspective of resolvability. In particular, chlorhexidine, berberine, usnic acid, and curcumin admit resolving sets of size 2, ellagic acid and epigallocatechin gallate require resolving sets of size 3, while colistin has metric dimension 5. These values reflect differences in the extent of branching, cyclic interaction, repeated local patterns, and structural symmetry present in the corresponding molecular graphs.

From the viewpoint of chemical graph theory, the significance of these results lies in the fact that metric dimension measures how efficiently the atomic positions of a molecular graph can be distinguished by distance information. Accordingly, it provides a mathematically precise indicator of distance-based structural uniqueness. Nevertheless, metric dimension does not encode the full chemical nature of a molecule, and different compounds may share the same metric dimension despite substantial differences in ring systems, substituent distribution, or functional groups. Therefore, it is most appropriately interpreted as a complementary topological descriptor rather than a complete structural or biological characterization.

The present work demonstrates that metric resolvability can offer useful structural insight into anti-biofilm compounds and can contribute to graph-based molecular analysis, indexing, and future descriptor-based studies. At the same time, broader chemical conclusions require investigations on larger and more diverse molecular datasets. Since exact computation of metric dimension is computationally challenging in general, such an extension is beyond the scope of the present paper and is being pursued in our ongoing work. Future studies may focus on large-scale molecular comparisons, algorithmic estimation of metric dimension, and the combined use of resolvability parameters with other graph-theoretic and physicochemical descriptors in QSPR/QSAR modeling.

## Data Availability

All the data used to finding the results is included in the manuscript.
